# Identification of Zinc Efficiency-Associated Loci (*ZEAL*s) and Candidate Genes for Zn Deficiency Tolerance of Two Recombination Inbred Line Populations in Maize

**DOI:** 10.3390/ijms23094852

**Published:** 2022-04-27

**Authors:** Jianqin Xu, Xiaoxin Qin, Zhongfu Ni, Fanjun Chen, Xiuyi Fu, Futong Yu

**Affiliations:** 1 Key Laboratory of Plant-Soil Interaction (MOE), Centre for Resources, Environment and Food Security, College of Resources and Environmental Sciences, China Agricultural University, Beijing 100193, China; xujqcau@163.com (J.X.); qinxx@pku.edu.cn (X.Q.); caucfj@cau.edu.cn (F.C.); 2 State Key Laboratory for Agrobiotechnology, Key Laboratory of Crop Heterosis and Utilization (MOE), Key Laboratory of Crop Genetic Improvement, China Agricultural University, Beijing 100193, China; nizf@cau.edu.cn; 3 Key Laboratory of Maize DNA Fingerprinting and Molecular Breeding, Maize Research Center, Beijing Academy of Agriculture & Forestry Science (BAAFS), Beijing 100097, China; fuxiuyicau@163.com

**Keywords:** zinc (Zn), Zn deficiency tolerance, quantitative trait locus (QTL), candidate gene, maize (*Zea mays* L.)

## Abstract

Zinc (Zn) deficiency is one of the most common micronutrient disorders in cereal plants, greatly impairing crop productivity and nutritional quality. Identifying the genes associated with Zn deficiency tolerance is the basis for understanding the genetic mechanism conferring tolerance. In this study, the K22×BY815 and DAN340×K22 recombination inbred line (RIL) populations, which were derived from Zn-inefficient and Zn-efficient inbred lines, were utilized to detect the quantitative trait loci (QTLs) associated with Zn deficiency tolerance and to further identify candidate genes within these loci. The BLUP (Best Linear Unbiased Prediction) values under Zn-deficient condition (-Zn) and the ratios of the BLUP values under Zn deficient condition to the BLUP values under Zn-sufficient condition (-Zn/CK) were used to perform linkage mapping. In QTL analysis, 21 QTLs and 33 QTLs controlling the Zn score, plant height, shoot and root dry weight, and root-to-shoot ratio were detected in the K22×BY815 population and the DAN340×K22 population, explaining 5.5–16.6% and 4.2–23.3% of phenotypic variation, respectively. In addition, seventeen candidate genes associated with the mechanisms underlying Zn deficiency tolerance were identified in QTL colocalizations or the single loci, including the genes involved in the uptake, transport, and redistribution of Zn (*ZmIRT1*, *ZmHMA*s, *ZmNRAMP6*, *ZmVIT*, *ZmNAS3*, *ZmDMAS1*, *ZmTOM3*), and the genes participating in the auxin and ethylene signal pathways (*ZmAFBs*, *ZmIAA17*, *ZmETR*, *ZmEIN2*, *ZmEIN3*, *ZmCTR3*, *ZmEBF1*). Our findings will broaden the understanding of the genetic structure of the tolerance to Zn deficiency in maize.

## 1. Introduction

Zinc (Zn) deficiency is a common micronutrient disorder in cereal plants, reducing crop productivity and nutritional quality. Zn has been reported to be deficient in 30% of the agricultural soils worldwide [1], and 50% of cereal crops are cultivated on soils with low plant-available Zn [2]. Compared with the demand of plants, the concentration of free Zn ions in the rhizosphere is usually very small, and it is quickly depleted without the proliferation from other pools, leading to the expression of Zn deficiency in crop plants [3,4]. The low level of Zn in the soil restricts the growth of crops and impairs the quantity and quality of crops. Maize is grown on around 160 million hectares worldwide [5], which provides about 30% of the food calories to more than 4.5 billion people in 94 developing countries [6]. As the second largest maize consumer and producer in the world, China was responsible for 22% of global maize output from 2012 to 2014 [7]. Although, in the intensive production systems of China, the maize yields (10t ha^−1^) are higher than the world average. However, as a limiting factor, Zn deficiency in soil causes farmers to achieve only 48–56% of the yield potential [8,9]. Maize is sensitive to Zn deficiency, and Zn-deficient symptoms are often observed in plants growing on the calcareous soil in the field. Zn deficiency reduces pollen viability and leads to pollen sterility in maize, and then to low kernel numbers [10]. Moreover, the insufficient assimilation caused by Zn deficiency has led to a shorter duration of linear grain-filling in later-growing kernels [11]. Because of the close relation of Zn flow among the soil–crop–human continuum, the purpose of some studies is to develop new cultivars with a strong genetic capacity to tolerate Zn deficiency. In this study, we identified important genes by exploiting the genetic variation of maize to provide the theoretical basis for developing new cultivars.

Quantitative trait locus (QTL) analysis provides an effective means of dissecting the genetic basis of complex traits in plants [12], and the basis for devising plant breeding strategies [13,14], greatly facilitating crop improvement. Zn deficiency is one of the most widespread limiting factors to crop production; however, only little effort in molecular works has been made so far to improve Zn efficiency in crop plants. The genetic basis for Zn deficiency tolerance in crop plants is poorly understood, since a majority of research has only focused on the QTLs associated with the Zn concentration and accumulation in grains [15,16,17]. Only very few studies have reported the performance under Zn-deficient condition, merely limited to rice [18,19], wheat [20], and navy bean [21]. The first study reported QTL associated with the performance under Zn deficiency in crop plants is conducted in rice [18]. This study identified QTLs associated with the most severe and susceptible Zn deficiency symptoms of rice in the field, including leaf bronzing and plant mortality, and biomass reduction in Zn-deficient condition. The two most influential QTLs controlling plant mortality were detected on chromosome 2 and explained 16.6% and 24.2% of the variation [18]. In wheat, Genc et al. [20] (2009) found that most of the QTLs linked to wheat seedling growth under Zn deficiency were associated with height genes. Combining bi-parental QTL mapping and genome-wide association analysis, Lee et al. [19] (2017) identified one putative candidate gene within the chromosome 6 QTL, which was associated with grain yield and component traits in both analyses. As for maize, almost all research has paid attention to the molecular basis of biofortification for mineral elements, especially iron and zinc [22,23,24]. However, there are very few reports on mapping Zn efficiency-associated loci (*ZEAL*s) in maize [25], which strongly hampers the development of untangling the internal mechanisms underlying the tolerance to Zn deficiency in maize. 

Plants have established the sophisticated mechanisms in resistance to Zn deficiency, including uptake from the environment as well as influx and efflux of Zn ions across adjacent cells, which require specific transmembrane transporters. Among these transporters, the zinc-regulated/iron-regulated transporter-like proteins (ZIPs) and heavy metal ATPases (HMAs) family play essential roles in the mechanisms on Zn deficiency tolerance in plants. Most studies concentrate on the *ZIP* and *HMA* gene families in *Arabidopsis* [26,27,28], rice [29,30,31,32], and barley [33,34]. However, there were only a few studies characterizing *ZIP*s and *HMA*s in maize [35,36,37,38]. 

In order to dissect the genetic basis in Zn deficiency tolerance in maize, in this study, the K22×BY815 and DAN340×K22 RIL populations were grown hydroponically under Zn-deficient and Zn-sufficient conditions. Utilizing QTL colocalizations mapped in different populations, important loci and the candidate genes associated with the tolerance to Zn deficiency in maize were explored.

## 2. Results

### 2.1. Phenotypic Variation in Zn Deficiency Tolerance

**Figure 1:**
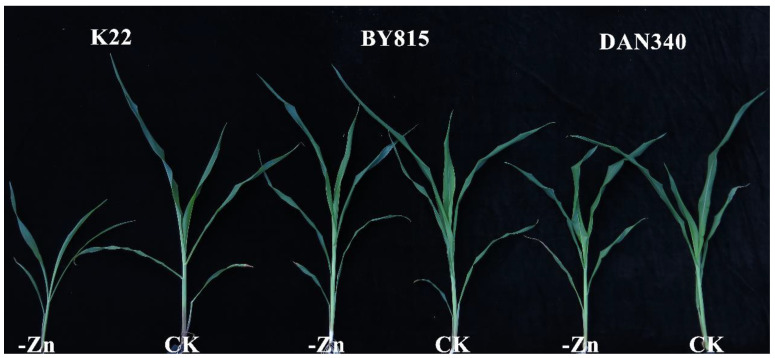
Under the Zn-deficient (-Zn: 0.3 μmol L^−1^ Zn-EDTA) and Zn-sufficient (CK: 10 μmol L^−1^ Zn-EDTA) conditions, maize inbred lines K22, BY815, and DAN340 (from left to right) were grown hydroponically for 21 days after transplanting. Representative shoots are displayed.

Under Zn-deficient condition, parents BY815 and DAN340 exhibited no visual Zn-deficient symptoms (Figure 1). K22 showed severe symptoms of Zn deficiency, including stunted growth, yellowish-white necrotic lesions on leaves, the necrosis of the leaf margins, and smaller leaves. By contrast, BY815 and DAN340 displayed no phenotypic differences between Zn-deficient and Zn-sufficient conditions. Regardless of treatments, all the traits associated with the tolerance to Zn deficiency showed significant differences between maternal and paternal parents (Table 1 and Table 2). BY815 had high Zn efficiencies (ZEs) based on the shoot (116%) and root (111%) dry weights, which were 6.0-fold and 4.8-fold higher than those of K22, respectively. Under Zn-deficient condition, the shoot and root dry weights of BY815 were more than five times higher than K22 (Table 1). Zn efficiencies based on the shoot and root dry weights of DAN340 were 4.1-fold and 3.8-fold higher than those of K22 (Table 2), respectively. The root-to-shoot ratios (R/S) for the parents increased in response to Zn deficiency, especially for K22.

**Figure 2:**
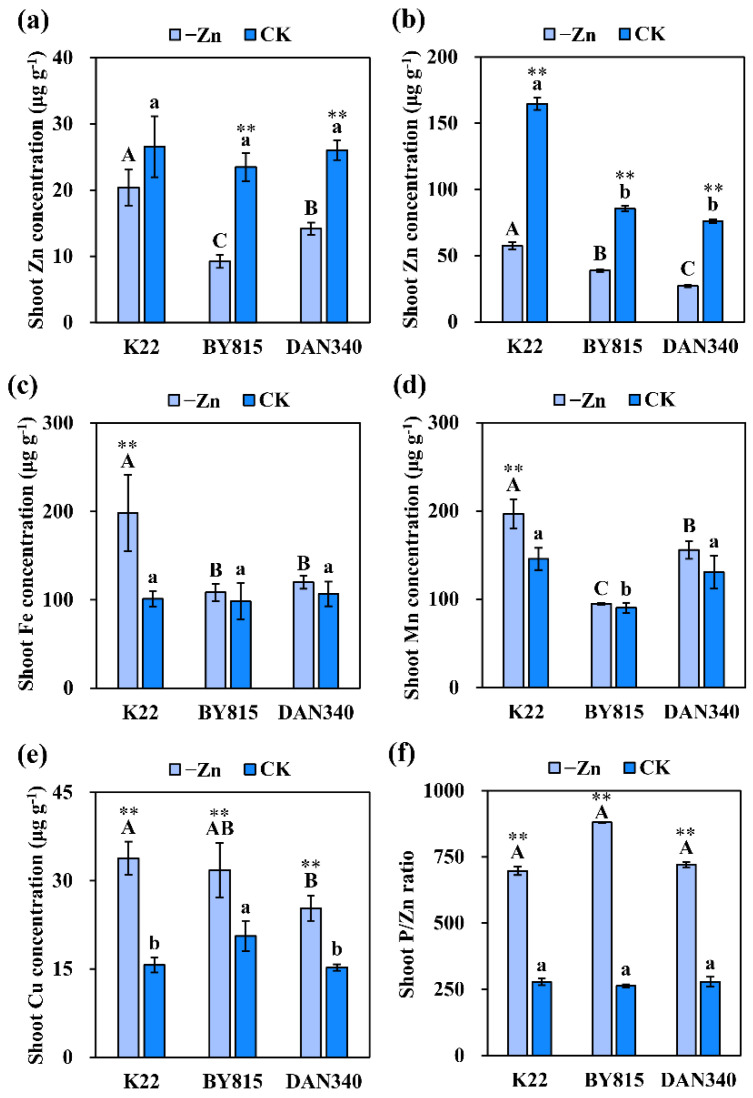
Shoot (**a**) and root (**b**) concentrations (μg g^−1^) of Zn, shoot concentrations of Fe (**c**), Mn (**d**), and Cu (**e**), and shoot P/Zn ratios (**f**) of parent plants (K22, BY815, and DAN340) hydroponically grown under Zn-deficient (0.3 μmol L^−1^ Zn-EDTA) and Zn-sufficient (10 μmol L^−1^ Zn-EDTA) conditions for 21 days after transplanting. Different uppercase (lowercase) letters indicate significant differences among K22, BY815, and DAN340 in the -Zn (CK) treatments at *p* ˂ 0.05. ** indicates significant differences between the -Zn and CK treatments at *p* ˂ 0.01.

Since shoot Zn concentrations of K22, BY815, and DAN340 under Zn deficiency were no more than 20 μg g^−1^, plants in the -Zn treatment were diagnosed to be Zn deficient. Deficiency in Zn substantially decreased Zn concentrations in the shoots and roots of BY815 and DAN340 by 60.8% and 54.7%, 45.5% and 64.4%, respectively (Figure 2a,b). However, Zn deficiency had no significant effects on the shoot Zn concentration of K22 (Figure 2a). In addition, Zn concentration of the sensitive inbred line K22 was 2.2-fold and 1.4-fold higher than those of tolerant inbred lines BY815 and DAN340, respectively, indicating that Zn-efficient genotypes may not necessarily have higher shoot Zn concentrations when compared with Zn-inefficient genotypes. Furthermore, our previous results confirmed that Zn efficiencies based on shoot and root dry weights were not correlated with Zn concentrations in the shoot and root of twenty maize inbred lines which contained K22, BY815, and DAN340 [25]. Zn deficiency significantly enhanced shoot concentrations of iron (Fe) and manganese (Mn) for K22 by 96.2% and 34.9%, respectively, whereas it had no effects on Fe and Mn concentrations of BY815 and DAN340 (Figure 2c,d). By contrast, shoot copper (Cu) concentration and P/Zn ratio for each parent were markedly increased by Zn deficiency (Figure 2e,f).

**Figure 3:**
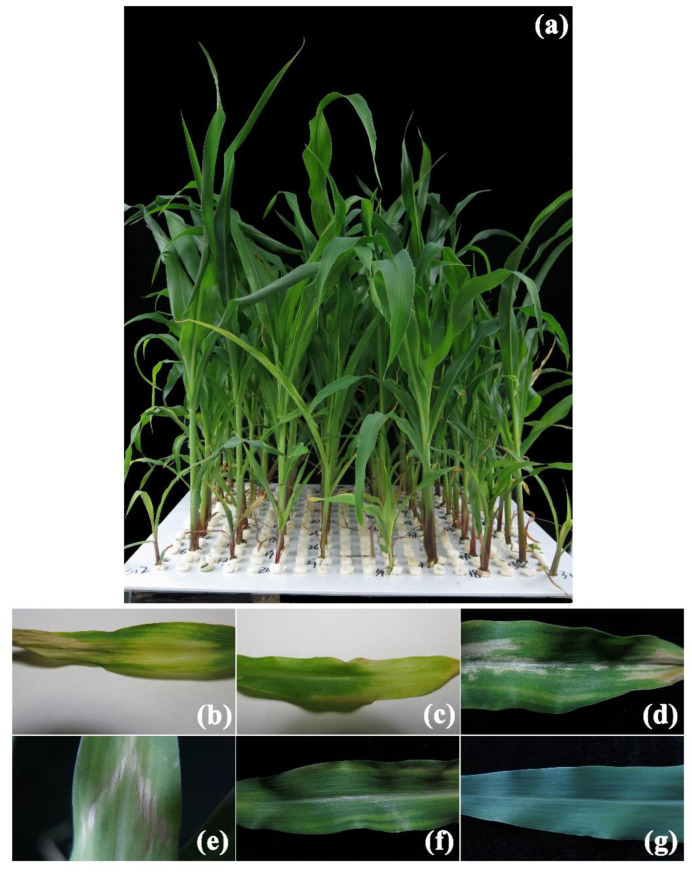
Phenotypic variations in the RILs and six scales for Zn score under Zn deficiency in maize. (**a**) In the -Zn treatment, two plants for each parent and 53 RILs were grown in hydroponics for 21 days after transplanting in each container. Large variations of Zn-deficient symptoms were observed among RILs. Zn score for each plant has been visually recorded for three times since 15th day after transplanting. (**b**) All leaves show wrinkled leaf margins and small malformed leaves, and more than 50% of areas on middle and young leaves show chlorosis and turn pale yellow. The whole leaf with obvious Zn-deficient symptoms tends to be dead with declines in physiological functions. (**c**) Clustering and malformed leaves are prominent symptoms, and 30–50% of areas on middle and young leaves show Zn deficient chlorosis with necrotic patches distributed on margins and tips of leaves. (**d**) All leaves are clustering and small leaves, and 20–30% of areas on middle and young appear brown patches. Moreover, Zn-deficient chlorosis has been improved compared with plants in score-0 and 1. (**e**) The stretch for all leaves of score-3 plant is strongly inhibited and necrotic spots are shown on 10–20% of areas in middle and young leaves. (**f**) There are no obvious differences of score-4 plant between the -Zn and CK treatments. However, banded chlorosis is still shown on leaf margins of middle and young leaves. (**g**) Score-5 plants are green and healthy without any Zn-deficient symptoms.

Large variations of Zn-deficient symptoms were observed among RILs (Figure 3a). For each trait, the mean in each line was calculated by at least three replications from the uniform seedlings. The mean values for each trait of the K22×BY815 and DAN340×K22 RIL populations were between maternal and paternal parents (Table 1 and Table 2; Figures S1 and S2). All traits associated with Zn deficiency tolerance exhibited abundant diversity among lines. The Coefficient of Variation (CV) for each trait among all RILs was calculated using the ratio of the mean to the standard error, ranging from 19.6% to 55.4%. All traits displayed normal distributions except for Zn score (Figures S1 and S2). The broad-sense heritability for each trait varied from 64.7–86.8%, suggesting that a great proportion of phenotypic variation in each trait was genetically controlled (Table 1 and Table 2). 

**Figure 4:**
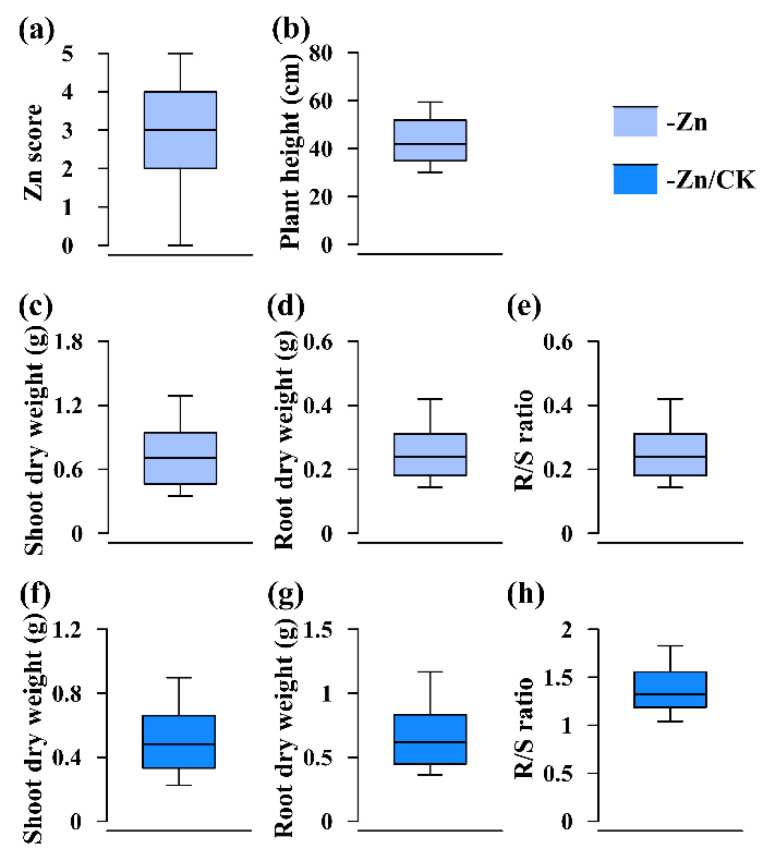
Phenotypic distribution of each trait in the K22×BY815 RIL population under different conditions (-Zn, -Zn/CK). (**a**) Zn score; (**b**) plant height; (**c**,**f**) shoot dry weight; (**d**,**g**) root dry weight; (**e**,**h**) R/S ratio. The solid line in each box represents the median values. The upper and bottom lines represent the 90th and 10th percentiles, respectively. The top and bottom edge of each box represent the 75th and 25th percentiles, respectively.

**Figure 5:**
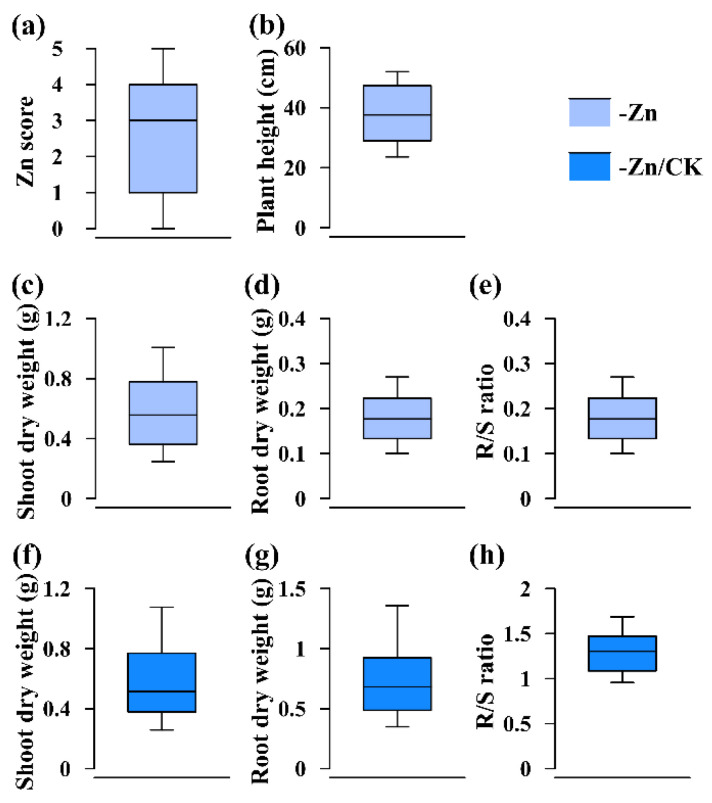
Phenotypic distribution of each trait in the DAN340×K22 RIL population under different conditions (-Zn, -Zn/CK). (**a**) Zn score; (**b**) plant height; (**c**,**f**) shoot dry weight; (**d**,**g**) root dry weight; (**e**,**h**) R/S ratio. The solid line in each box represents the median values. The upper and bottom lines represent the 90th and 10th percentiles, respectively. The top and bottom edge of each box represent the 75th and 25th percentiles, respectively.

**Figure 6:**
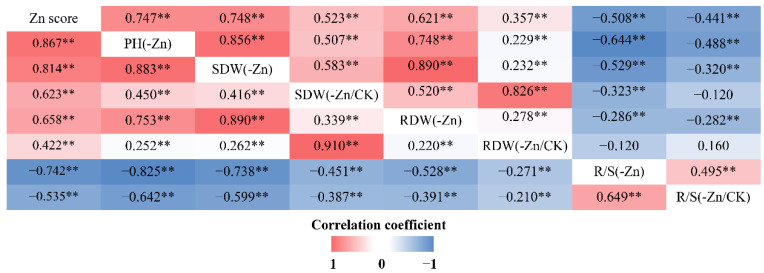
Correlation coefficients among eight traits in the K22×BY815 (upper triangular matrix) and DAN340×K22 (lower triangular matrix) RIL populations. PH, plant height; SDW, shoot dry weight; RDW, root dry weight; R/S, root-to-shoot ratio. **, *p* < 0.01.

Zn score, which was to assess the ability to tolerate Zn deficiency and was ranked from 0 to 5 (Figure 2b–g, Figure 4a and Figure 5a), had the highest broad-sense heritability (Table 1 and Table 2). As shown in Figure 4 and Figure 5, the 75th percentiles of shoot and root dry weights in the -Zn/CK treatment in these two RIL populations were no more than 1, indicating that shoot and root biomass accumulation in most of the RILs were reduced in response to Zn deficiency. However, the 90th percentiles of R/S ratio in the -Zn/CK treatment were higher than 1 (Figure 4e,h and Figure 5e,h), suggesting that the root-to-shoot ratios of the RILs responded positively to low Zn stress. In these two RIL populations, the Zn score was significantly correlated with plant height, shoot and root dry weights, and R/S ratio using Spearman correlation analysis (*p* < 0.01). Furthermore, significant correlation was found among any other two traits using Pearson correlation analysis (*p* < 0.01) (Figure 6).

### 2.2. QTL Detection

**Figure 7:**
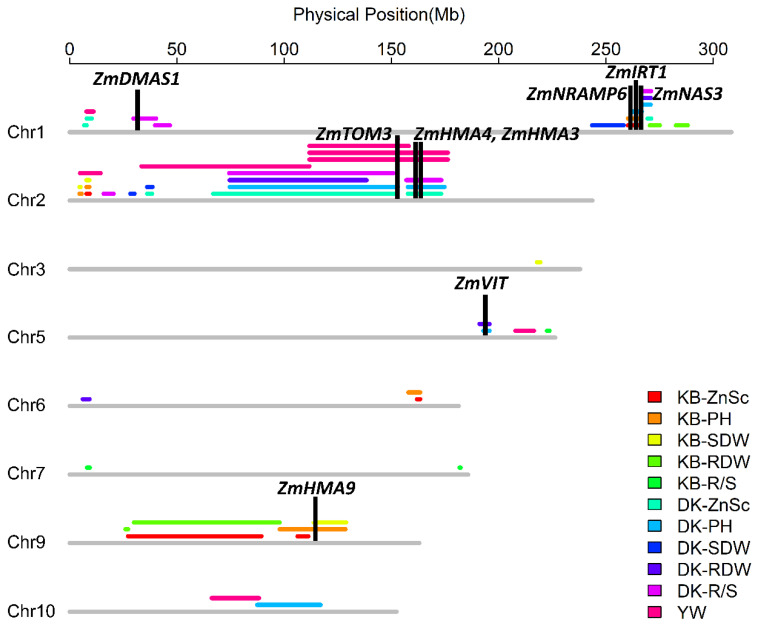
Fifty-four Zn efficiency-associated loci (*ZEAL*s) detected on chromosome 1, 2, 3, 5, 6, 7, 9, 10 in the K22×BY815 (KB) and DAN340×K22 (DK) RIL populations, and the QTL colocalizations identified in the Ye478×Wu312 (YW) RIL population [25]. Candidate genes considered to be associated with Zn deficiency uptake, transport, and redistribution (*ZmDMAS1*, *ZmTOM3*, *ZmNRAMP6*, *ZmIRT1*, *ZmHMA3* and *ZmHMA4*, *ZmHMA9*, and *ZmVIT*) are depicted in black columns.

In the K22×BY815 RIL population, based on the empirical threshold of the LOD score for each trait, a total of 21 QTLs were identified in the -Zn and -Zn/CK treatments (Figure 7, Table S1). Five QTLs (*qKB-ZnSc1-1*, *qKB-ZnSc2-1*, *qKB-ZnSc6-1*, *qKB-ZnSc9-1*, and *qKB-ZnSc9-2*) controlling Zn score were identified on chromosome 1, 2, 6, and 9, together explained 39.5% of phenotypic variation. Alleles from BY815 at four QTLs except for *qKB-ZnSc1-1* had an additive effect of 0.31–0.42 for increased Zn score. Five QTLs for plant height were mapped on chromosome 1, 2, 6, and 9, explaining 5.5–12.0% of phenotypic variation. The allele associated with increased plant height at the four QTLs, except for *qKB-PH1-1* on chromosome 1, came from BY815. The second largest-effect QTL, *qKB-PH2-2*, that accounted for 12.0% of phenotypic variation, was located between PZE-102017472 and SYN18069. Four loci (*qKB-SDW2-1*, *qKB-SDW2-2*, *qKB-SDW9-1*, and *qKB-SDW3-1*) controlling shoot dry weight were identified on chromosome 2, 3, and 9. At the three QTLs (*qKB-SDW2-1*, *qKB-SDW2-2*, and *qKB-SDW9-1*) on chromosome 2 and 9, the allele from BY815 increased the shoot dry weight by 0.09–0.13 g. Four QTLs associated with the root dry weight were detected on chromosome 1 and 9, explaining 5.9–11.2% of phenotypic variation. The allele from K22 at two loci (*qKB-RDW1-1*, *qKB-RDW1-2*) on chromosome 1 had additive effects for the increased root dry weight. In addition, the allele that increased the ZE based on root dry weight at two loci (*qKB-RDW9-1*, *qKB-RDW9-2*) on chromosome 9 came from BY815. The third largest-effect QTL was mapped between PZE-109023988 and PZE-109025227 on chromosome 9, explaining 11.2% of phenotypic variation. Three QTLs (*qKB-R/S5-1*, *qKB-R/S7-1*, and *qKB-R/S7-2*) controlling the R/S ratio under Zn deficiency were identified on chromosome 5 and 7, explaining 6.4% of phenotypic variation. Major QTL *qKB-R/S7-2*, located in the genetic interval of 136.9–138.9 cM on chromosome 7, explained 16.6% of phenotypic variation in the K22×BY815 RIL population. 

In the DAN340×K22 RIL population, the BLUPs in the -Zn and -Zn/CK treatments were used to perform the QTL mapping, and 33 QTLs were detected based on the empirical threshold of the LOD score for each trait (Figure 7, Table S2). Six QTLs (*qDK-ZnSc1-1*, *qDK-ZnSc1-2*, *qDK-ZnSc1-3*, *qDK-ZnSc2-1*, *qDK-ZnSc2-2*, and *qDK-ZnSc2-3*) for the Zn score were identified on chromosome 1 and 2, elucidating 4.5–23.3% of phenotypic variation. Among them, *qDK-ZnSc2-2*, a QTL was located to the interval flanked by PZE-102080558 and PZE-102113254 (66.9–152.5 Mb) on chromosome 2 and accounted for 23.3% of the phenotypic variation observed for Zn score. The QTL *qDK-ZnSc2-3*, located in the interval of 157.7–173.2 Mb on chromosome 2, explained 21.2% of phenotypic variation. The allele from DAN340 at two QTLs had additive effects of 0.66–0.69 for enhanced Zn score. Six QTLs (*qDK-PH1-1*, *qDK-PH1-2*, *qDK-PH2-1*, *qDK-PH2-2*, *qDK-PH5-1*, and *qDK-PH10-1*) controlling plant height were detected on chromosome 1, 2, 5, and 10, explaining 4.2–15.5% of phenotypic variation. Eight QTLs that were associated with the shoot dry weight were mapped on chromosome 1, 2, 3, and 10, explaining 4.8–23.2% of phenotypic variation. Among them, the second largest-effect loci, *qDK-SDW2-1*, was identified between PZE-102084401 and PZE-102113254 on chromosome 2, explaining 23.2% of phenotypic variation. The allele which increased the shoot dry weight under Zn deficiency by 0.16 g at this locus, was provided by DAN340. The remaining seven loci had relatively minor effects, with LOD scores ranging from 3.4 to 6.5. For the root dry weight, six QTLs were detected on chromosome 1, 2, 5, and 6, accounting for 8.9–14.2% of phenotypic variation. Seven QTLs controlling the R/S ratio were detected on chromosome 1 and 2 and accounted for 6.6–21.2% of phenotypic variation. Among these seven loci, *qDK-R/S2-1* and *qDK-R/S2-2*, which had large effects with PVEs of 20.5% and 21.2%, respectively, were detected in the intervals of 74.4–151.0 Mb and 156.9–173.3 Mb, respectively. Another five QTLs controlling R/S ratio contributed to 6.6–11.7% of phenotypic variation.

In summary, the number of QTLs per trait varied from three to eight in these two RIL populations. Support intervals determined by the 1-LOD method in the KB and DK population averaged 5.1 cM (10.4 Mb) and 4.7 cM (17.4 Mb), with a range from 2.0 to 13.6 cM (0.4–67.9 Mb) and a range from 0.8 to 12.3 cM (1.1–85.6 Mb), respectively. The explained phenotypic variation (PVE) of each QTL in the KB and DK populations varied from 5.5% (*qKB-PH6-1*) to 16.6% (*qKB-R/S7-2*) and from 4.2% (*qDK-PH5-1*) to 23.3% (*qDK-ZnSc2-2*), respectively (Tables S1 and S2). In these two RIL populations, 14.8% of 54 single loci had a PVE ≥ 15%, suggesting that quite a few QTLs with higher PVEs and a number of QTLs with lower PVEs mainly contribute to the genetic component of the traits associated with Zn deficiency tolerance. This also reflects the complex basis of the tolerance to Zn-deficiency stress in maize. Additionally, 71.4% (15/21) of the identified QTLs in the KB population had additive effects for increasing the values of the detected traits, and this value in the DK population was 60.6% (20/33). These results implied that parent lines BY815 and DAN340 have occupied abundant favorable alleles, which may be available for the improvement of Zn deficiency tolerance during maize breeding.

### 2.3. QTL Colocalization and Candidate Genes Identification

Genomic regions overlapped by the QTLs controlling the same traits and colocalizations of the QTLs detected by different the RIL populations are important for the identification of candidate genes. Evidence for identifying candidate genes by the QTLs colocalized by the loci detected in different populations have been recorded by multiple studies in maize [14,39,40]. Apart from the QTLs detected in the KB and DK populations in this study, we also used the QTLs identified in the Ye478×Wu312 (YW) RIL population to further determine the QTL colocalization among different populations [25]. Five QTL colocalization intervals were identified in eleven QTLs detected by different traits in the KB, DK, and YW RIL populations: two localizations on chromosome 1 and three localizations on chromosome 2 (Figure 7, Table 3). 

Based on the annotated genes in the B73 reference genome Version 5, a total of 253 candidate genes was identified within these QTL colocalizations. According to the functional descriptions of 253 candidate genes in *Arabidopsis* and rice on the MaizeGDB Database (Available online: http://www.maizeGDB.org (accessed on 15 January 2022)) and the Gramene Database (Available online: https://www.gramene.org (accessed on 15 January 2022)) (Table S3), *ZmIRT1* (Zm00001eb052440) and *ZmNRAMP* (Zm00001eb051790), which were considered to be associated with Zn deficiency tolerance, were identified within the overlapped interval of *qKB-PH1-1* and *qDK-PH1-1*, which both controlled plant height under Zn deficiency. In addition, another seven genes which may be associated with the responses to Zn deficiency, containing *ZmTOM3* (Zm00001eb093430), *ZmHMA3* (Zm00001eb095020), *ZmHMA4* (Zm00001eb095010), *ZmCTR3* (Zm00001eb096080), *ZmVIT* (Zm00001eb248740), *ZmIAA17* (Zm00001eb258220), and *ZmHMA9* (Zm00001eb389830), were identified within other overlapped regions colocalized by the QTLs controlling different traits (Table 4). Beyond that, according to previous studies on Zn deficiency in plants, eight genes associated with Zn uptake and translocation, stress sensing and signaling were identified within the intervals of five single loci, containing *ZmDMAS1* (Zm00001eb010040), *ZmNAS3* (Zm00001eb052890), *ZmETR* (Zm00001eb054170), *ZmEIN2* (Zm00001eb054060), *ZmEIN3* (Zm00001eb331080), *ZmEBF1* (Zm00001eb011850), and *ZmAFBs* (Zm00001eb085030, Zm00001eb421340) (Table 4).

## 3. Discussion

### 3.1. Physiological Mechanisms Underlying ZE

Zn efficiency, which is considered to characterize the tolerance to Zn deficiency, can be defined as the ability of a genotype to grow and yield well under Zn-deficient condition for a standard cultivar. A linear relationship between the reduction in shoot dry matter and the severity of leaf symptoms of Zn deficiency are observed in a range of cereals [41], and some reports further confirmed that the visual symptoms of Zn deficiency are significantly correlated with Zn efficiency [42,43]. Consistent with previous results mentioned above, Zn scores evaluating the symptoms of Zn deficiency in the linkage population for the present study were significantly correlated with R/S ratios, as well as the absolute and relative values of the dry matter weights of the shoots and roots (*p* < 0.01) (Figure 6). Therefore, the visual scoring system has provided a rapid selection criterion to estimate Zn efficiency at the seedling stage.

Our results showed that the root-to-shoot ratios of most RILs were increased in response to low Zn stress, further confirming that the increase in R/S ratio occurs as an initial response to Zn deficiency in crops [44,45]. Zn-efficient and Zn-inefficient inbred lines displayed differential responses to Zn deficiency in shoot and root growth. Root growth can be increased in some cereals as a consequence of Zn deficiency [46]. For a Zn-efficient inbred line like BY815, which had the highest ZEs based on the shoot and root dry weights (116% and 111%), the root dry matter increased more than the shoot and may be attributed to the change in the partitioning of the dry matter, enabling higher nutrient uptake [47]. A Zn-efficient inbred line like DAN340, with higher ZEs (below 100%), reduced the shoot growth at the expense of the root growth, so as to decrease the metabolic demands of the shoots and the greater relative root surface area for ion absorption [46,48]. Furthermore, there was significant correlation among the R/S ratio, the shoot and root dry weights (Figure 6). Therefore, the absolute and relative shoot and root biomass accumulation can be suitable indicators to characterize Zn efficiency in the linkage populations in maize.

In our work, Zn-efficient genotypes did not have higher Zn concentrations in the shoot or root at a low level of Zn supply. A number of studies have indicated that plant tissue Zn concentration is not a dependable parameter for evaluating differential Zn efficiency among genotypes [49,50]. Zn concentrations in tissues do not reflect how much is physiologically available for metabolic processes, or how much is inactivated or compartmented in nonmetabolic pools [51]. Zn efficiency in crop plants does not differ in shoot concentrations and may be due to internal biochemical utilization or the subcellular compartmentation of Zn in leaf cells [52,53]. Compartmental analysis of ^65^Zn efflux from bean leaves provides further evidence in support of compartmentation in one of the ZE mechanisms, which indicates that the Zn-efficient genotype not only has moderately more Zn in the cytoplasm, and less Zn in the vacuole, but also exhibits a faster Zn exchange from vacuole when compared with the Zn-inefficient genotype [54].

Fe, Mn, and Cu concentrations in the shoots of Zn-deficient plants increased likely due to competitive interaction of Zn deficiency-inducible transport proteins in the transport of Fe, Mn, and Cu compared to Zn across the plasma membrane [34,55]. In addition, maize inbred lines accumulated high amounts of P in shoots on deficient Zn supply, resulting in a higher P/Zn ratio in the shoots. This is completely in accord with the general view of many researchers, that P has an antagonistic effect on Zn uptake by plants and the degree of the increase in the P/Zn ratios in plant tissue is indicative of the degree of Zn-deficiency stress in plants [56,57]. Moreover, this effect is possibly regulated by the expression of the genes encoding P transporters, which is induced by the plant’s Zn nutritional status [58,59]. Zn deficiency not only results in increased expression of the genes encoding high-affinity P uptake transporters in the roots [60], but also increases the expression of P transporters involving in the transport of P to the xylem, leading to enhanced P transport to the shoot [61].

### 3.2. Comparisons of QTLs Identified in this Study with Previous Reports

To our knowledge, very few Zn efficiency-associated loci have been identified in maize, but numerous QTLs were identified to be associated with Zn and other microelements concentrations in maize [22,24,62,63,64,65]. In addition, there were lots of QTLs related to the traits we used in this study, such as plant height, shoot or root dry weight, and the R/S ratio under other conditions rather than Zn-deficient condition [66,67,68,69]. Thereby, these QTLs in previous studies were compared with current results based on their physical position. 

In total, 41 of 54 QTLs for different traits in two linkage populations have been identified to be colocalized with the QTLs reported by other researchers (Tables S1 and S2). Among these QTLs, 21 QTLs were also identified in the QTLs controlling mineral concentrations in grain, including Zn, Fe, Mn, and P concentrations, detected in previous studies [22,24,62,63,64,65]. Especially, the major-effect QTL *qKB-R/S7-2* in the KB population was colocalized with two QTLs for Zn and Fe concentrations of the kernel [62]. In addition, five QTLs with a PVE > 20% mapped on chromosome 2 in the DK population were colocalized with the loci controlling Fe and Mn concentrations of the grain detected by Gu et al. [22] (2015) and Zhang et al. [63] (2017). These results imply that these colocalized QTL regions may have pleiotropic effects on the mineral concentration of grains and seedlings in maize.

Moreover, 35 of 54 QTLs in the current study were identified in the QTLs associated with plant height, the shoot or root dry weight, the R/S ratio, and the seedling root traits in previous reports (Tables S1 and S2). Both *qKB-PH6-1* and *qKB-PH9-1*, controlling plant height under Zn deficiency on chromosome 6 and 9, were overlapped with the loci related to plant height reported by Zhang et al. [69] (2018) and Luo et al. [68] (2017), respectively. This indicates that these regions may contain a single gene exerting a strong effect underlying the QTL colocalization for plant height under Zn-deficiency stress. Three QTLs (*qKB-RDW1-1*, *qKB-RDW1-2*, and *qDK-RDW1-1*) on chromosome 1, two QTLs (*qDK-RDW2-1* and *qDK-RDW2-2*) on chromosome 2, one QTL on chromosome 5 (*qDK-RDW5-1*) and 6 (*qDK-RDW6-1*), and three QTLs (*qKB-RDW9-1*, *qKB-RDW9-2* and *qDK-RDW9-1*) on chromosome 9, which were associated with root dry weight, were colocalized with the QTLs regulating the seedling root traits, including the root length, root surface area, and root volume under normal condition [64,70], the axial root number, and the primary root length under high- and low-nitrogen levels [67]. These findings suggest that these QTLs may harbor several genes with pleiotropic effects on root traits at the seedling stage.

### 3.3. Candidate Genes Associated with Zn Uptake and Transport

Zm00001eb052440, also known as *ZmIRT1*, which belongs to the *ZIP* family, was mapped in the QTL colocalization overlapped by *qKB-PH1-1* and *qDK-PH1-1*, controlling plant height under Zn-deficient condition in two RIL populations (Figure 7, Tables S1 and S2). It is reported that the yeast mutants expressing *ZmIRT1* showed the strongest propagation under both Zn- and Fe-limited conditions [35]. The *ZmIRT1*-overexpressing *Arabidopsis* plants not only exhibit increased levels of Zn and Fe in different tissues, but also show altered tolerance to various Fe and Zn conditions compared with wild-type plants [71]. 

Actually, IRT1 has been identified to be a metal transporter with a broad substrate specificity [26,72]. A novel yeast uptake assay based on an inductively coupled plasma–mass spectrometry analysis of 31 different metal and metalloid ions proved that the HvIRT1 protein is able to transport Zn^2+^ and Cd^2+^, in addition to Fe^2+^ [73]. The broad substrate specificity of HvIRT1 is similar to that found for AtIRT1 [27,74]. For another homolog, *OsIRT1*, its transcripts are induced by Fe, Zn, Cu, and Mn deficiencies, and over-expressing plants are sensitive to excess Zn and Cd [75]. Moreover, *OsIRT1*-overexpressing plants accumulate more Fe and Zn in the shoots, roots, and mature seeds [75]. This indicates that OsIRT1 may be a transporter for Zn. 

Additionally, *ZmHMA3* and *ZmHMA4* were also identified in the QTL colocalization detected by three QTLs (Table 4). Up to now, many *HMA* genes have been identified and studied in *Arabidopsis* and rice. *ZmHMA3* and *ZmHMA4* are the homologs of *AtHMA3* and *OsHMA3*, and *ZmHMA9* is the ortholog of *OsHMA9* [38]. The overexpression of *AtHMA3* leads to the enhancement of Zn accumulation in both shoots and roots [76]. A ^67^Zn-labeling experiment and a mobility experiment indicate that OsHMA3 is important for Zn detoxification and storage by sequestration into the vacuoles in the roots [77,78].

These findings suggest that *ZmHMA3* and *ZmHMA4* may be involved in the transport of Zn ions. Zm00001eb051790 (*ZmNRAMP6*), a member of the *NRAMP* family in maize, was detected in the overlapped region colocalized by *qKB-PH1-1 and qDK-PH1-1* in two different RIL populations. NRAMP proteins play important roles in Zn, Fe, Mn, and Cd transport across the cellular membranes in living organisms [79]. Additionally, the conserved metal-binding site methionine dictates substrate preference in Nramp family divalent metal transporters [80]. The high expression of *NRAMP3* and *NRAMP4* genes is found in the leaves of Zn/Cd hyperaccumulating *Arabidopsis halleri* [81]. AtNRAMP4 is an identified Zn transporter [82], and *AtNRAMP4* expression modulates the concentrations of Zn^2+^, Cd^2+^, and Mn^2+^ in roots [83]. In addition, the candidate gene Zm00001eb248740 encoding vacuolar iron transporter has been identified in this research (Figure 7). It is reported that rice VIT1 and VIT2 function to transport Fe^2+^, Zn^2+^, and Mn^2+^ into the vacuoles in yeast [84]. OsVIT1 and OsVIT2 are suggested to play a role in Fe/Zn translocation between source and sink organs [84]. Beyond that, three candidate genes identified within a single locus were associated with chelation mechanisms in the uptake and translocation of Zn, containing *ZmNAS3* (Zm00001eb052890), *ZmDMAS1* (Zm00001eb010040), and *ZmTOM3* (Zm00001eb093430). On the one hand, nicotianamine synthase (NAS) catalyzes the biosynthesis of nicotianamine (NA), which can chelate Zn ions to form stable complexes Zn(II)-NA [85]. On the other hand, deoxymugineic acid (DMA) is synthesized from NA via nicotianamine aminotransferase (NAAT) and DMA synthase (DMAS) [86,87,88,89]. Phytosiderophores (PSs), including NA and DMA, secreted by the transporter of mugineic acid (TOM) proteins [90,91,92], can form Zn complexes that are as stable as Fe(III)-PS [93], including Zn(II)-DMA [94] and Zn(II)-NA [95]. These stable complexes, especially Zn(II)-DMA, which is the preferred form for the uptake and long-distance transport of Zn, display important roles in Zn absorption from the soil and the distribution within plants [96,97,98].

### 3.4. Candidate Genes Linked with Hormone Signaling

Reactive oxygen species (ROS), which should be scavenged to keep cellular turgor and structures actively functioned [99,100], accumulate under Zn-deficient condition, leading to the oxidative degradation of IAA, and then the repression in the shoot growth [101]. Zn plays an important role in the production of indole-3-acetic acid [102,103], and Zn deficiency adversely affects the synthesis of the growth regulating compounds such as auxins, resulting in decreases of production and activity of indole-3-acetic acid [104]. Zn deficiency signals may be linked with hormones that include auxin and ethylene, which would indirectly regulate downstream Zn-responsive genes, such as the genes responsible for Zn acquisition, uptake, and transport, and the genes controlling Zn chelator biosynthesis and release [105].

Zm00001eb421340, also known as *ZmAFB*, is identified to encode an auxin signaling F-box protein (AFB) and is involved in auxin-dependent regulation via the interaction between TIR1/AFB with Aux/IAA proteins in plants. Zm00001eb258220, which was mapped within *qKB-R/S5-1* and *qZEAL-RDW5-1* on chromosome 5, encodes a member of the *Aux/IAA* gene family (*ZmIAA17*). Exogenous auxin influences plant root growth by regulating the expression of early auxin-responsive genes of auxin/indole-3-acetic acid (*Aux/IAA*), which may be quickly activated and transcribed after being processed by auxin [106,107,108,109]. *OsIAA9*, an ortholog of *ZmIAA17*, is greatly induced by exogenously applied auxin [110]. Ectopic overexpression of *OsIAA9* results in fewer crown and lateral roots and reduces the inhibition of root elongation by auxin, suggesting that *OsIAA9* is a negative regulator of auxin-regulated root growth [111]. 

In addition, another five candidate genes probably involved in the ethylene signaling pathway were identified in this study, containing *ZmETR* (Zm00001eb054170), *ZmCTR3* (Zm00001eb096080), *ZmEIN2* (Zm00001eb054060), *ZmEIN3* (Zm00001eb331080), and *ZmEBF1* (Zm00001eb011850) (Table 4, Figure 7). Zn is required for ethylene response because it is a component of the ethylene receptor [112]. Several ethylene signal genes are found in some plants to be regulated by salt stress, Zn stress, and Fe deficiency stress [113,114,115,116]. 

## 4. Materials and Methods

### 4.1. RIL Population

K22×BY815 (KB) and DAN340×K22 (DK) RIL populations, constructed by Xiao et al. [117] (2016), consist of 209 and 192 lines and are derived from the cross between maternal parent K22 and paternal parent BY815, maternal parent DAN340 and paternal parent K22, respectively. The genetic map of the K22×BY815 RIL population contains 2263 SNPs which cover 1670.4 cM throughout the genome. The genetic map of the DAN340×K22 RIL population includes 2100 SNPs that cover 1698.4 cM throughout 10 chromosomes. Average intervals of adjacent SNPs for K22×BY815 and DAN340×K22 RIL populations are 0.74 cM and 0.81 cM, respectively. 

### 4.2. Plant Culture in Hydroponics

Maize seeds were sterilized for 30 min in a 10% solution of H_2_O_2_, washed with distilled water, and soaked in saturated CaSO_4_ for 10 h, and then germinated on moist filter paper in the dark at room temperature. Two days later, the germinated seeds were wrapped in moist filter paper roll and grown. At the stage of two visible leaves, the seedlings were selected and transferred into a 40 L black tank (665 mm × 410 mm × 160 mm, length × width × height). Plants were cultured in hydroponics under Zn-deficient (-Zn: 3 × 10^−4^ mmol L^−1^ Zn-EDTA) and Zn-sufficient conditions (CK: 1 × 10^−2^ mmol L^−1^ Zn-EDTA). Each treatment contained three replicates. For each treatment, 53 RILs and two seedlings of each parent were grown in each tank, as shown in Figure 3a, and four tanks which contained all RILs were considered as a replication in each treatment. For each treatment, a total of twelve tanks was used as three replicates in each RIL population. Two experiments were conducted for two RIL populations randomized in incomplete blocks. 

The adjusted Hoagland nutrient solution contained (mmol L^−1^): 0.5 NH_4_NO_3_, 0.5 CaCl_2_, 1.5 Ca(NO_3_)_2_, 0.75 K_2_SO_4_, 0.65 MgSO_4_, 0.1 KCl, 0.25 KH_2_PO_4_, 1.0 × 10^−3^ H_3_BO_3_, 0.35 EDTA-Fe(II), 8.0 × 10^−3^ CuSO_4_, 1.2 × 10^−2^ MnSO_4_, 4.0 × 10^−5^ (NH_4_)Mo_7_O_24_, 4.0 × 10^−3^ NiCl. Solution pH was set at 5.5–6.0. Nutrient solution was renewed every three days and aerated by a pump. Maize seedlings were cultured in hydroponics in a growth chamber with strictly controlled conditions: 28 °C during 14 h light period from 8:00 to 22:00, 22 °C during 10 h dark period, average light intensity with 350 μmol m^−2^ s^−1^ that were measured at canopy.

### 4.3. Phenotyping Methods

Zn-deficient symptoms appeared since the 9–12th day after transplanting, and the Zn score (ZnSc) for each plant was visually recorded three times since 15th day after transplanting. Six scales (0–5) were designed to assess the tolerance to Zn deficiency. Zn deficiency tolerance scoring mainly depends on the suppression of the growth and development for the whole plant, as well as the areas of chlorosis and necrotic patches distributed on middle and young leaves.

Score 0: plant develops about three leaves with one sprout, plant growth is stunted, and leaf elongation is severely suppressed. All leaves show wrinkled leaf margins and small malformed leaves, and more than 50% of areas on middle and young leaves appear chlorosis and turn pale yellow. The whole leaf with obvious Zn-deficient symptoms tends to be dead, with declines in physiological functions (Figure 2b). Score 1: four leaves and one sprout have been developed, and plant height and leaf elongation are strongly suppressed, as the score-0 plant is. Clustering and malformed leaves are prominent symptoms, and 30–50% of areas on middle and young leaves show Zn-deficient chlorosis with necrotic patches distributed on the margins and tips of leaves (Figure 2c). Score 2: plant has 5 leaves and 1 sprout, exhibiting similar malformations in plant development and distorted leaf growth as with the score-1 plant. All leaves are clustering and small leaves, and 20–30% of areas on middle and young appear brown patches. Moreover, Zn-deficient chlorosis has been improved compared with plants in score-0 and 1 (Figure 2d). Score 3: suppression in plant height and internode length is markedly decreased when compared with the score-2 plant. However, the stretch for all leaves is strongly inhibited and the necrotic spots are shown on 10–20% of areas in middle and young leaves (Figure 2e). Score 4: there are no significant differences in plant growth and development between the -Zn (Zn-deficient condition) and CK treatment (Zn-sufficient condition). However, banded chlorosis is still shown on the leaf margins of the middle and young leaves (Figure 2f). Score 5: plants are green and healthy (Figure 2g). 

The experiment was terminated at the 21st day after transplanting, and the plant heights (PH) were measured first, then the shoots (SDW) and roots (RDW) were stored in the envelopes separately. All samples were dried at 75 °C until a constant weight, then shoot and root dry weights were recorded separately, and the R/S ratios (R/S) were calculated. Additionally, the values of SDW, RDW, and R/S in the -Zn/CK treatment refer the ratios of the values under Zn-deficient condition (-Zn) to the values under Zn-sufficient condition (CK). Dried shoots and roots for parent samples were separately ground into fine powder, and 0.3000 g powder was digested with HNO_3_-H_2_O_2_ in a microwave accelerated reaction system (CEM, Matthews, NC, USA). The concentrations of Fe, Mn, Cu, Zn and P in the digested solutions were determined by inductively coupled plasma atomic emission spectroscopy (ICPAES, OPTIMA 3300 DV, Perkin-Elmer, Waltham, MA, USA). The ratio of shoot P content to shoot Zn content for each plant was calculated as P/Zn ratio.

### 4.4. Statistical Analysis

Means of different inbred lines were compared using one-way ANOVA at a 0.05 level of probability by SPSS 20.0 (SPSS, Chicago, IL, USA). The phenotypic difference between two parents for each RIL population was calculated by Student’s *t*-test. The linear mixed effect function lmer in the lme4 package of R was fitted to each RIL in the RIL population to obtain the BLUP (Best Linear Unbiased Prediction) value for each trait: *y_ijk_ = μ + G_i_ + E_j_ + G_i_ × E_j_ + R(E)_jk_ +* *ε_i_*, where *y_ijk_* is the phenotypic value of *i*th inbred line in the *j*th environment and *k*th replication, *μ* is the overall mean, *G_i_* is the genetic effect of the *i*th inbred line, *E_i_* is the environmental effect of *j*th environment, *Gi* × *Ej* is the interaction between genotype and environment for the *i*th inbred line and *j*th environment, *R(E)_jk_* is the *k*th replication within the *j*th environment, and *ε_ijk_* is the residual error. The broad-sense heritability for each trait was estimated using the formula: *H*^2^ *= σ_G_*^2^*/(σ_G_*^2^ *+ σ_GE_*^2^*/e +σ_E_*^2^*/re),* where *σ_G_*^2^ is genetic variance, *σ_GE_*^2^ is the interaction of genotype and environment, *σ_E_*^2^ is residual error, while *e* and *r* are the number of environments and replications, respectively.

### 4.5. QTL Mapping

In this study, the BLUP values under Zn-deficient condition (-Zn) and the ratios (-Zn/CK) of the BLUP values under Zn-deficient condition to the values under Zn-sufficient condition were used to perform the QTL analysis. Here, the ratio of -Zn to CK for each trait was calculated using the BLUPs for each trait from the -Zn treatment and CK, respectively. The identification of QTL was performed using composite interval mapping (CIM) [118], implemented in the Windows QTL Cartographer version 2.5 (N.C. State University, Bioinformatics Research Center, Raleigh, NC, USA). The scanning interval between markers was set at 0.5 cM, and the window size was set at 10 cM. Model 6 of the Zmapqtl module was selected for detecting position and additive effects of QTL. The threshold logarithm of odds (LOD) values in this study were estimated by permutation tests with minimum of 1000 replicates at a significance level of *p* = 0.05. The support interval of the QTL position was determined using the 1-LOD interval method (1 LOD away from the peak LOD value). Moreover, the QTL having the same support intervals were defined as an identical QTL.

### 4.6. Annotation of Candidate Genes

According to the physical distance of peak bins, genes within the refined QTL peak and their functional descriptions were identified using the maize B73 reference genome assembly Version 5, available on the MaizeGDB Database (Available online: http://www.maizeGDB.org/ (accessed on 15 January 2022)) and the Gramene Database (Available online: https://www.gramene.org/ (accessed on 15 January 2022)). The functions of candidate genes were further confirmed by the annotations of the orthologs in *Arabidopsis* and rice.

## Figures and Tables

**Table 1:** Phenotypic variations of the traits associated with Zn deficiency tolerance in the parental lines K22 and BY815, and K22×BY815 RIL population.

Trait *^a^*	Treatment *^a^*	Parents				RIL Population	
		K22♀	BY815♂	*^b^*	Mean	CV (%)	*H*^2^ (%)*^c^*
ZnSc	-Zn	1	3	**	2.5	55.4	83.1
PH (cm)	-Zn	29.5	67.2	**	41.8	26.1	72.4
SDW (g)	-Zn	0.29	1.88	**	0.79	46.6	81.2
SDW	-Zn/CK	0.23	1.16	**	0.53	50.2	NA
RDW (g)	-Zn	0.12	0.64	**	0.28	41.0	74.8
RDW	-Zn/CK	0.29	1.11	**	0.64	47.2	NA
R/S	-Zn	0.34	0.37	**	0.34	19.6	64.7
R/S	-Zn/CK	1.31	1.12	*	1.26	21.9	NA

*^a^* ZnSc, PH, SDW, RDW, and R/S represent Zn score, plant height, shoot dry weight, root dry weight, and R/S ratio, respectively. -Zn and -Zn/CK indicate the data under Zn-deficient condition and the ratio of the data under Zn-deficient condition to the data under Zn-sufficient condition. The same as below. *^b^* * and ** indicate significant differences between K22 and BY815 at *p* < 0.05 and *p* < 0.01, respectively. *^c^* NA indicates not available data. The same as below.

**Table 2:** Phenotypic variations of the traits associated with Zn deficiency tolerance in the parental lines DAN340 and K22, and DAN340×K22 RIL population.

Trait	Treatment	Parents			RIL Population		
		DAN340♀	K22♂	*^a^*	Mean	CV (%)	*H*^2^ (%)
ZnSc	-Zn	3	1	**	2.4	53.1	86.8
PH (cm)	-Zn	59.4	31	**	37.5	30.2	81.5
SDW (g)	-Zn	1.04	0.36	**	0.61	50.1	84.1
SDW	-Zn/CK	0.8	0.26	**	0.54	53.8	NA
RDW (g)	-Zn	0.27	0.13	**	0.18	37.1	77.4
RDW	-Zn/CK	0.86	0.31	*	0.56	48.7	NA
R/S	-Zn	0.36	0.33	**	0.34	29.0	72.2
R/S	-Zn/CK	1.16	1.34	**	1.17	23.9	NA

*^a^* * and ** indicate significant differences between DAN340 and K22 at *p* < 0.05 and *p* < 0.01, respectively.

**Table 3:** QTLs colocalized with the loci controlling different traits in the K22×BY815, DAN340×K22, and Ye478×Wu312 RIL populations.

Chr	Interval (Mb) *^a^*	QTL	Trait *^b^*	Physical Interval (Mb) *^a^*	LOD	R^2^ (%)	RIL Population *^c^*
1	261.4–265.2	*qKB-PH1-1*	PH	260.2–265.2	5.7	10.3	KB
		*qDK-PH1-1*	PH	261.4–266.6	9.5	14.2	DK
1	270.4–270.8	*qKB-RDW1-1*	RDW	270.4–275.1	3	5.9	KB
		*qDK-RDW1-1*	RDW	267.1–270.8	6.4	11.4	DK
2	7.9–9.4	*qKB-ZnSc2-1*	ZnSc	7.9–9.4	4.5	7.8	KB
		*qZEAL-ZnSc2-1*	ZnSc	4.9–14.4			
2	36.2–38.4	*qDK-ZnSc2-1*	ZnSc	36.2–38.4	10	16.4	DK
		*qZEAL-ZnSc2-2*	ZnSc	33.5–111.8	3.9	10.1	YW
2	108.6–134.3	*qDK-SDW2-1*	SDW	74.6–153.0	12.4	23.2	DK
		*qZEAL-SDW2-1*	SDW	111.8–176.3	6.1	20.7	YW
		*qZEAL-SDW2-2*	SDW	111.8–176.3	6.3	17.3	YW

*^a^* The position refers to the B73 reference sequence Version 5. *^b^* ZnSc, PH, SDW, RDW represent Zn score, plant height, shoot and root dry weight, respectively. *^c^* KB, DK and YW represent the K22×BY815, the DAN340×K22, and the Ye478×Wu312 RIL populations, respectively.

**Table 4:** The information of candidate genes within the loci.

QTL	Chr	Gene ID	Gene Position (bp) *^a^*	Description
*qDK-R/S1-3*	1	Zm00001eb010040	31742085–31745237	ZmDMAS1—Deoxymugineic acid synthase 1
*qDK-R/S1-3*	1	Zm00001eb011850	38561776–38563168	ZmEBF1—EIN3-binding F box protein 1
*qKB-PH1-1, qDK-PH1-1*	1	Zm00001eb051790	262493944–262496834	ZmNRAMP—Metal transporter Nramp 6
	1	Zm00001eb052440	264886590–264888963	ZmIRT1—Iron-regulated transporter 1
*qDK-PH1-1*	1	Zm00001eb052890	266321457–266323150	ZmNAS3—Nicotianamine synthase 3
*qKB-RDW1-1*	1	Zm00001eb054060	270891072–270897163	ZmEIN2—Ethylene-insensitive protein 2
*qKB-RDW1-1*	1	Zm00001eb054170	271172369–271177408	ZmETR—Signal transduction histidine kinase hybrid-type ethylene sensor
*qDK-ZnSc2-2*	2	Zm00001eb085030	67296998–67301921	ZmAFB—Auxin signaling F-box
*qDK-ZnSc2-2, qDK-PH2-1, qDK-SDW2-1, qDK-R/S2-1*	2	Zm00001eb093430	152901305–152906124	ZmTOM3—Transporter of mugineic acid 3
*qDK-ZnSc2-3, qDK-PH2-2, qDK-R/S2-2*	2	Zm00001eb095010	162889727–162893463	ZmHMA4—Cadmium/Zinc-transporting ATPase HMA4
	2	Zm00001eb095020	162910876–162914856	ZmHMA3—Cadmium/Zinc-transporting ATPase HMA3
*qDK-ZnSc2-3, qDK-PH2-2, qDK-R/S2-2*	2	Zm00001eb096080	170130733–170150672	ZmCTR3—Constitutive triple response 3
*qDK-PH5-1, qDK-RDW5-1*	5	Zm00001eb248740	193961138–193962096	ZmVIT—Vacuolar iron transporter (VIT) family protein
*qKB-R/S5-1, qZEAL-RDW5-1*	5	Zm00001eb258220	222819625–222821061	ZmIAA17—auxin-responsive Aux/IAA family member
*qKB-R/S7-2*	7	Zm00001eb331080	182932191–182934361	ZmEIN3—Ethylene insensitive 3 family protein
*qKB-PH9-1, qKB-ZnSc9-2, qKB-SDW9-1*	9	Zm00001eb389830	114577311–114585730	ZmHMA9—Heavy metal translocating P-type ATPase transports
*qDK-PH10-1*	10	Zm00001eb421340	113391837–113396456	ZmAFB—Auxin signaling F-box

*^a^* The position refers to the B73 reference sequence Version 5.

## Data Availability

The data presented in this study are available on request from the corresponding author.

## Supplementary Materials

The following supporting information can be downloaded at: https://www.mdpi.com/article/10.3390/ijms23094852/s1.
