# Supplementary material for: Identification of Zinc Efficiency-Associated Loci (ZEALs) and Candidate Genes for Zn Deficiency Tolerance of Two Recombination Inbred Line Populations in Maize
Source: Int J Mol Sci. 2022 Apr 27;23(9):4852. doi: 10.3390/ijms23094852 (PMC9106061; doi:10.3390/ijms23094852)
Supplement: Supplementary file 1 [file ijms-23-04852-s001.zip › Supplementary Material S1 (Figure S1-S2).pdf]

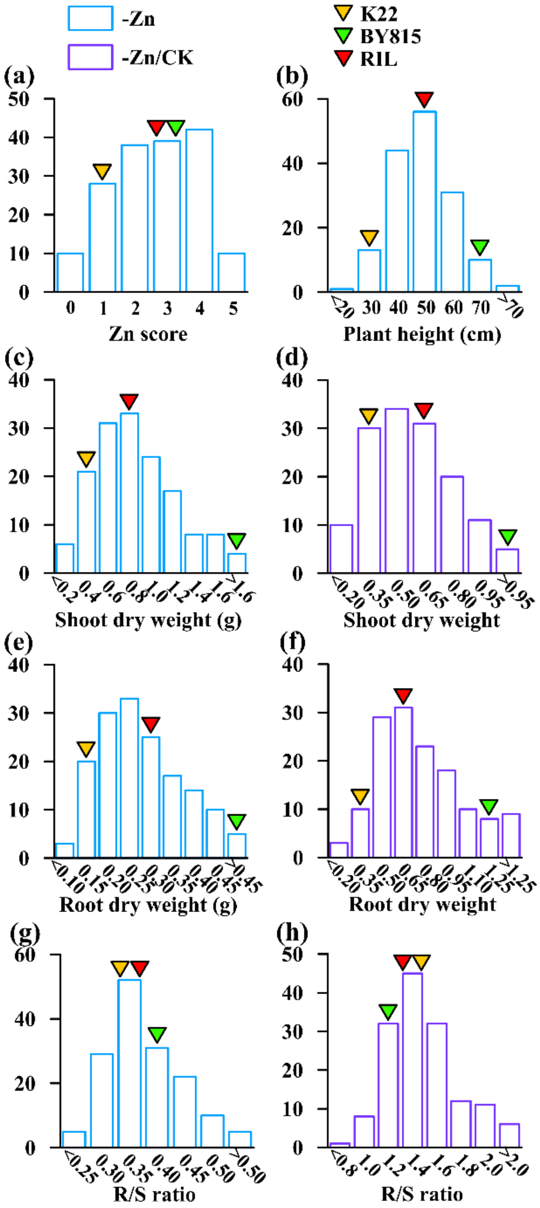

Figure S1 Distribution of Zn score (a), plant height (b), shoot (c, d) and root (e, f) dry weight, R/S ratio (g, h) in the K22×BY815 RIL population in the -Zn and -Zn/CK treatments. Yellow and green triangles represent the mean values for the traits of Zn-inefficient parent K22 and Zn-efficient parent BY815, respectively. Red triangles indicate the means values of the traits in the RIL population.

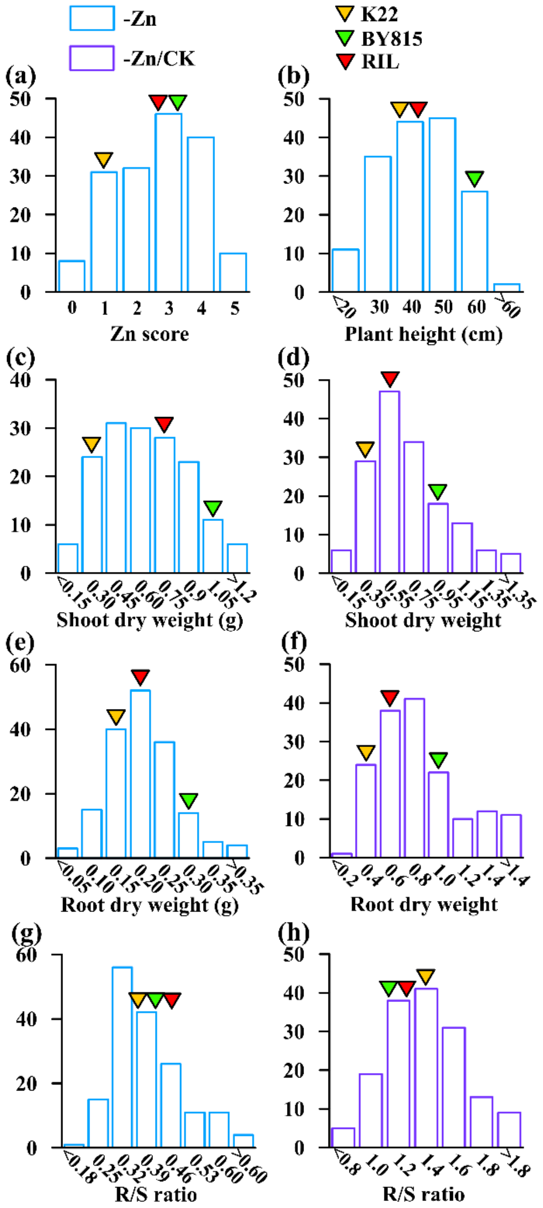

Figure S2 Distribution of Zn score (a), plant height (b), shoot (c, d) and root (e, f) dry weight, R/S ratio (g, h) in the DAN340×K22 RIL population in the -Zn and -Zn/CK treatments. Yellow and green triangles represent the mean values for the traits of Zn-inefficient parent K22 and Zn-efficient parent DAN340, respectively. Red triangles indicate the means values of the traits in the RIL population.
